# Exploring Laser-Induced Breakdown Spectroscopy as a Potential Tool in Mohs Micrography: A Mini Review

**DOI:** 10.7759/cureus.1842

**Published:** 2017-11-13

**Authors:** Sandeep Singh, Rupak Desai, Mitul Modi, Showket Hussain

**Affiliations:** 1 Neurology, Institute of Human Behavior and Allied Sciences (IHBAS), Delhi, India; 2 Research Coordinator, Atlanta Veterans Affairs Medical Center; 3 PGY 1 Ap/cp, Department of Pathology, Pennsylvania Hospital of University of Pennsylvania, Philadelphia & Gujarat Cancer and Research Institute, Ahmedabad, India; 4 Department of Molecular Oncology, National Institute of Cancer Prevention and Research

**Keywords:** mohs micrographic surgery, laser induced break down spectroscopy, skin cancer, basal cell carcinoma, spectroscopy, artificial neural network, melanoma, laser, cancer detection

## Abstract

Mohs micrographic surgery is the technique of surgically removing skin tumors by gradually excising thin layers and visualizing under a microscope till a tumor-free zone is obtained. During the surgical procedure, visible tumors are surgically removed. During the second stage, if tumor margins are clear with the positive specimen at depth, only depth cavitations need to be done without altering the tumor diameter. Defining the depth during this procedure is a major challenge due to the nonexistence of proper guidelines. Using the laser-induced breakdown spectroscopy (LIBS) technique, depth profiling can be performed precisely, preventing excessive tissue removal and reducing time consumption during the microscopic examination.

## Introduction and background

**Figure 1:**
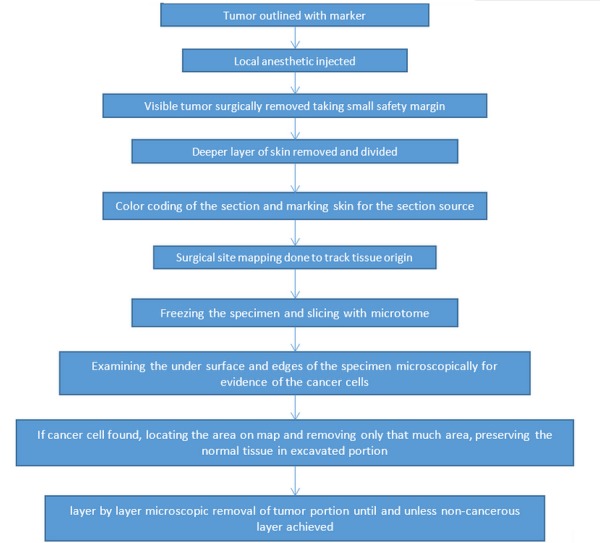
Algorithm Approach for the Mohs Procedure

Mohs surgery is the technique of surgically removing skin tumors by gradually excising thin layers and visualizing under a microscope till a tumor-free zone is obtained. For skin cancers, it is the most accepted and efficient technique of removing tumors. The traditional treatment approach involves removal of affected areas allowing for a safe margin around and below the tumor. The removed specimen is sent to pathology for histopathological reporting, which requires at least two weeks. Only after the report is available can it be finalized whether the patient is clear of the tumor or a positive margin is still remaining. If a positive margin persists, a further procedure is required to remove the remaining tumor. This overall process is tedious for the patient and decreases patient compliance [1]. Here, Mohs surgery plays a critical role in ensuring precise excision via microscopic examination during surgery, leading to the removal of the last root eliminating the chance of missing any cancerous tissue. The algorithm approach for the Mohs procedure is depicted in Figure 1 [2]. 

## Review

Overview of laser-induced breakdown spectroscopy technique

Laser-induced breakdown spectroscopy (LIBS) is a novel spectroscopic technique for elemental analysis based on optical emission following of a sample using laser pulse [3]. The spectra release from the plasma contains information about the elemental constituents of the tissue [4]. Elemental line spectra generated in femto- to nano-seconds ablating nanograms of material makes the whole procedure nearly minimally destructive [5-6]. Studies show that there occurs a difference in the trace mineral milieu of normal cells when they become cancerous [6-8]. This concept can be used to differentiate normal tissue from cancerous tissues. Such variations can be detected using the LIBS technique even in the parts-per-million (PPM) concentration very easily. Studies have suggested its various uses in biological material evaluation [6, 9-12]. LIBS' depth profiling ability has been shown in various studies and can be used for in-situ analysis of the samples [13]. The approach is used for studying gallstone samples that contain different layers, providing valuable information regarding their nucleation. Exponential decreases in the zinc concentration in human tissues have been used to detect tissues via the LIBS technique [14]. Studies have shown the varying concentration ratios of elements in cancerous and normal tissues, thus showing that LIBS may be used as a tool for cancer detection [15]. They established the real-time in-situ characterization of the normal and abnormal tissues without performing the traditional biopsy [16]. Certain studies have explored the possibility of using LIBS in in-vivo cancer detection [6-7, 17-18].

The postulation

Laser pulse will be administered in the center of the lesion on the skin. Vaporization of a nanogram or microgram of the sample will generate the plasma plum in a non-traumatic fashion in fractions of seconds. Optionally, the procedure may be carried out on the patient in real-time with local anesthesia [6, 17]. This plasma plum will be recorded by a photo spectrometer. Computer software using artificial neural network (ANN) techniques will be used to analyse the emitted spectral line based on the intensity and ratios of different trace elemental constituents of the tissue cell [6, 17]. After the depth profiling, tumors up to the certain depth will be removed and margins' positivity will be assessed using Mohs procedure. Now, the margin identified as positive will be targeted and the depth of that cancerous root will be defined by the LIBS procedure.

Use of LIBS during various steps of Mohs micrography

During the surgical procedure, visible tumors are surgically removed. During the second stage, if tumor margins are clear with the positive specimen at depth, only depth cavitations need be done without altering the tumor diameter. Defining the depth during this procedure is a major challenge due to the nonexistence of proper guidelines. This depth ranges from deep fascia / periosteum / subcutaneous fat at different sites [19-21]. Using LIBS technique, depth profiling can be done precisely, reducing excessive tissue removal and time consumption during the microscopic examination. During the second stage, margins need to be examined via microscopy and positive margins should be again analyzed by LIBS for the precise depth in the direction of possible growth. Tumors in challenging areas like the nose and face could involve multiple rounds, requiring a whole day. LIBS could conserve some time by demarcating the exact depth of the lesion to an extent.

Defining the margin before making the cut is crucial

Defining the depth and the size of the lesion are the two most important factors governing the prognosis. Tumors with a depth of more than 4 mm bear higher metastatic and recurrence probability. LIBS can improve diagnostic sensitivity for deep tumors which may be missed on microscopy. Defining the margin is a crucial step in skin tumor management, as studies have reported an increased rate of recurrence with incomplete excision [19-20]. Wider margins have better prognosis but are cosmetically unacceptable. The advisable excision margin is debatable, and it ranges from 2 to 15 mm in non-melanotic tumors and 15 mm to 26 mm for melanotic tumors [22]. There is one more challenge - margin distortion during tissue processing may prevent the pathologist from confidently defining the positive margin [23]. Such situations could lead to higher chances of missing positive tissue margins and creates the diagnostic dilemma of whether further surgical clearance should be done. Here, LIBS can be proven to be a beneficial tool in deciding the margin positivity of the doubtful lesions [24].

Advantages of LIBS combined with Mohs surgery

1. LIBS can provide Mohs surgeons a quick approach as it requires just a few seconds to ablate the site and provide the analysis in the spectral form.

2. Tumors extending deep into the vessels and nerves will require a baseline depth profiling before they could be removed, because if approached directly there might be a chance to injure the vessel or nerve blindly. LIBS will give a baseline depth up to which cancer is extending and then we could have a defined map up to what area or depth we need to cut the tumor [25].

3. Melanotic atypia sometimes requires special stains and permanent sections to avoid missing any malignant cells, prolonging the whole procedure. LIBS could be fruitful in such situations delineating the normal from abnormal cells [26].

4. In certain situations, inflammation obscures the tumor requiring an LIBS approach to scrutinize the tissue properly [27].

5. It can precisely predict the optimal margins consuming less time if combined with MOHS procedure [28-29]. 

## Conclusions

The review explores the use of LIBS as a potential technique for the depth profiling of tumors during Mohs micrographic surgery. The potential of real-time diagnosis of tumor margins and defining the extent of tumors could be a time-saving approach when used with the conventional Mohs micrographic surgery. However, it is not limitations free. The major limitation is the requirement of a LIBS probe which could go deep inside, as it would be difficult to assess samples with thickness more than 20 mm. There will also be the requirement for a probe which could collect spectral data in the horizontal plane, as these tumors don’t strictly go deep inside but also have side branches in the horizontal plane.
